# Mitochondria and chloroplasts function in microalgae energy production

**DOI:** 10.7717/peerj.14576

**Published:** 2022-12-16

**Authors:** Diego F. Gomez-Casati, Julieta Barchiesi, Maria V. Busi

**Affiliations:** CEFOBI-CONICET, Universidad Nacional de Rosario, Rosario, Santa Fe, Argentina

**Keywords:** Microalgae, Energy, Mitochondria, Chloroplasts

## Abstract

Microalgae are organisms that have the ability to perform photosynthesis, capturing CO_2_ from the atmosphere to produce different metabolites such as vitamins, sugars, lipids, among others, many of them with different biotechnological applications. Recently, these microorganisms have been widely studied due to their possible use to obtain clean energy. It has been postulated that the growth of microalgae and the production of high-energy metabolites depend on the correct function of cellular organelles such as mitochondria and chloroplasts. Thus, the development of different genetic tools to improve the function of these organelles is of high scientific and technological interest. In this paper we review the recent advances in microalgae engineering and the role of cellular organelles in order to increase cell productivity and biomass.

## Introduction

Currently, the energy demand *per capita* is increasing with the development of society. It is estimated that the population will be around 10,000 million people by 2050. Thus, humans as well as other organisms that live on earth, will have different problems such as global warming and energetic crisis (Alishah Aratboni et al., 2019). On the other hand, it was reported that the consumption of non-renewable energy sources such as fossil fuels represents approximately 86% of the energy consumption worldwide, which is also a serious problem for the environment (Sun et al., 2018). For this reason, the search for alternative energy sources that are efficient, competitive and renewable is of high scientific and economic interest. In this sense, microalgae could be key organisms for clean energy production.

These microorganisms have the ability to synthetize sustainable bioproducts and biofuels by using CO_2_ or another carbon source (Du & Benning, 2016; Vitova et al., 2015). The advantage of microalgae is that the solar energy is trapped in organic matter and potentially converted into different compounds such as starch, sugars, lipids, amino acids, vitamins, *etc*. (Sun et al., 2018). In addition, microalgae photosynthesis contributes to CO_2_ fixation, removing this greenhouse gas from the atmosphere, while the biomass produced can be converted into biofuels, different bioproducts and biobased chemicals (Chen et al., 2016). For this reason, microalgae are considered as a promising raw material for applications in biofuel production but also high-value bioproducts due to their high photosynthetic capacity, efficiency and its short life cycle. Compared to plants, microalgae have a higher capacity to fix CO_2_ and subsequently convert it into biomass. They can produce up to 60 times more fuel than land plants using the same units of surface area and sunlight, and they also have a reproductive capacity between five to ten-fold greater respect to several crops. On the other hand, microalgae have an advantage in terms of their impact on the environment, since they do not need to use resources such as soil. In addition, they do not require fertilizers, pesticides or clean water, since they can be cultivated in salt water or waste water (Skjanes, Rebours & Lindblad, 2013). Another benefit of microalgae is that they can use the CO_2_ generated by different industries to carry out photosynthesis. Thus, while microalgae generate energy, they contribute to the decarbonization of the atmosphere. For this reason, there is a growing interest from various industries in developing new strategies to improve microalgae performance; however, at the present, biotechnological applications of these organisms are limited due to the lack of understanding of carbon metabolism and energy conversion. Moreover, a bibliographic search for microalgae productivity and the role of mitochondria and chloroplasts has not been reported previously. The information we have collected and reviewed here will be of interest for researchers working in marine biology and biotechnology as well as those working in molecular biology, biochemistry and physiology of cell organelles and on genetic engineering of microorganisms.

## Survey methodology

The PubMed (https://pubmed.ncbi.nlm.nih.gov/) and Google Scholar (https://scholar.google.com/) repositories were used to search the following terms: mitochondria, microalgae and mitochondria, algae, respiration, microalgae and photosynthesis, chloroplasts, lipids and microalgae, hydrogen production in microalgae, microalgae engineering, microalgae editing, organellar crosstalk, algae productivity, algae biomass.

### Mitochondrial function, microalgae productivity and energy production

Mitochondria are double-membrane-bound organelles present in eukaryotic organisms. Depending on their physiological requirements, some cells may have about 10 to 1,000 mitochondria whit a proteome composed of more than 1,200 proteins. This organelle plays a critical role in different cellular processes such as energetic metabolism, carbon and nitrogen metabolism, thermoregulation, calcium and iron homeostasis, regulation of apoptosis, and in the synthesis of Fe-S and heme groups (Kotiadis, Duchen & Osellame, 2014; Ryan & Hoogenraad, 2007).

Mitochondrial biogenesis requires the correct function of the electron transport chain (mETC), which is composed by four multiprotein complexes (complexes I–IV) and an ATP synthase (Complex V) (Moller, Rasmusson & Van Aken, 2021), many of them formed by ferrosulfoproteins and hemoproteins (Gray, 1999; Gross & Bhattacharya, 2009; Poyton & McEwen, 1996). Fe-S proteins and hemoproteins participate in a wide variety of cellular processes such as many enzymatic reactions, respiration, cofactor biosynthesis, ribosome biogenesis, regulation of gene expression, and DNA and RNA metabolism in almost all organisms (Busi & Gomez-Casati, 2012; Gomez-Casati et al., 2021; Lill & Muhlenhoff, 2008).

Among other functions, microalgae are biocatalysts for the production of different compounds such as lipids, starch, cellulose, proteins and hydrogen. The ability of these organisms to convert light energy in many compounds of economic importance places them within the organisms with the greatest potential to produce clean energy (Elman et al., 2022; Nath et al., 2015). Thus, many efforts have been made to understand in more detail some processes and metabolic pathways that allow these photosynthetic organisms to supply different energy demands (Nath et al., 2015).

Particularly, bio-hydrogen production has attracted attention as a secondary energy carrier, because this compound is the lightest carbon-neutral fuel, has a high energy capacity per mass unit, and it is easy to store. In addition, it is a renewable energy source, and eco-friendly. Although various technologies are currently being developed for H_2_ production, at present none of them have been used as a replacement for traditional fuels (Nath et al., 2015). In this sense, the identification of more robust microalgae or the development of genetically modified strains for various biotechnological applications would be of great scientific interest.

There are other examples of genetic manipulation of microalgae in order to modify metabolic pathways and/or mitochondrial functions to improve their productivity. One of the approaches used was the alteration of lipid metabolism using a multiplexed CRISPR/Cas9 to modify enzymes belonging to the long chain acyl-CoA synthetase (LACS) family (Hao et al., 2022). Although the general role of LACS in other organisms is well known, little is known about how these enzymes regulate lipid metabolism in algae. The study of *Phaeodactylum tricornutum* knockout/knockdown strains of LACS, particularly the mutants deficient in LACS3, which has a mitochondrial localization, showed a deficiency in the accumulation of triacylglycerol (TAG), as well as an alteration in the fatty acid profile (Hao et al., 2022). This study demonstrated the importance of the proper function of mitochondria in the production of lipids in oleaginous microalgae.

It has been recently described in other organisms that mitochondrial stress would also lead to an increase in lipid accumulation (Jennings et al., 2021; Lee et al., 2013; Turchi et al., 2020). In addition, it was also reported that photoautotrophically or photoheterotrophically cultured stressed microalgae cells can accumulate large quantities of TAG in lipid bodies, reaching about 46–65% of dry weight (Banerjee, Dubey & Shukla, 2016; Du & Benning, 2016; Goold et al., 2015, 2016; Kwak et al., 2016; Li et al., 2010; Vitova et al., 2015). Furthermore, it was shown that the combination of two stress conditions also increased lipid production; however, the use of more than two different conditions (*i.e.*, nutrient limitation, temperature variation, pH, *etc*.) resulted in a lower growth rate of the algae cells. Based on these data, we propose that the induction of mitochondrial stress, caused, for example, by the deficiency of some enzyme of a key metabolic pathway in the organelle, in conjunction with the application of another type of stress (*i.e.*, nitrogen or phosphorus limitation) could substantially improve the accumulation of lipids, with an increase of the biomass and the productivity of microalgae.

Recently, a novel mechanism was also described in *C. reinhardtii*, which showed that it has the ability to modify the location of its mitochondria inside the cells. The positioning of cell organelles was associated with various metabolic and signaling functions in unicellular organisms. Under normal conditions, it was observed that mitochondria are randomly distributed in this microalga, and relocate to the cell periphery under low inorganic carbon levels (Harmon et al., 2022). This mechanism is not known in detail; however, it is believed to be related to a carbon concentration mechanism. It is of particular importance to identify some mutant strains where this organelle relocation does not occur in order to understand the role of mitochondria in this process and its relationship with energy production (Harmon et al., 2022). Thus, based on these data, it was proposed that mitochondria would have a fundamental but also a little-known role in the accumulation of lipids in microalgae and also in the production of H_2_, which would have important biotechnological implications.

### Crosstalk between mitochondria and chloroplasts in microalgae productivity

Various works have shown that the mitochondria have a key role in the provision of cellular energy, as well as in the participation in retrograde regulation mechanisms involved in the biogenesis of other organelles (*i.e.*, chloroplasts) in plants and algae (Busi et al., 2011; Liu & Butow, 2006; Liu et al., 2018). Although mitochondria and chloroplasts were traditionally considered as autonomous compartments, there is much evidence that the crosstalk between these organelles directly impacts on cell physiology (Raghavendra & Padmasree, 2003). For example, mitochondrial metabolism, particularly the bioenergetic reactions of oxidative electron transport and phosphorylation are still active in the light and are essential for maintain photosynthetic carbon assimilation (Busi et al., 2011; Raghavendra & Padmasree, 2003).

To achieve this goal, the correct function of the mETC and the photosynthetic machinery are essential. Numerous studies reported that mETC plays a crucial role in the retrograde regulation of the expression of different genes related to the photosynthetic process in different organisms (Liu et al., 2018; Matsuo & Obokata, 2006). Particularly, in *C. reinhardtii*, it was showed that the induction of photosynthetic genes responds to different signals from the mitochondrial and chloroplast electron transport chains (Matsuo & Obokata, 2006).

According to these results, Li et al. (2008) demonstrated that *Chlorella zonfingiensis* strains are able to grow and accumulate astaxanthin under dark conditions using glucose as the only source of carbon and energy. It is important to note that astaxanthin, a carotenoid from the terpene family, is a powerful antioxidant and colorant that has numerous applications in the nutraceutical, cosmetic and food industries (Guerin, Huntley & Olaizola, 2003). In this study, an increase in the levels of several chloroplastic enzymes such as β-carotenoid ketolase (BKT) and beta-carotenoid hydroxylase (CHYb) was observed in the presence of glucose analogs and glucosamine (hexokinase inhibitor) that led to an increase in astaxanthin amounts (Li et al., 2008). In contrast, the impairment of the mETC using some inhibitors such as antimycin A or rotenone partially decreased the transcription of those genes, while in the presence of salicyl hydroxamic acid (SHAM), an inhibitor of the alternative mitochondrial pathway, the transcription of such genes was completely inhibited, leading to lower astaxanthin levels. These data are in agreement with the involvement of mitochondria in the induction of the metabolic pathway that leads to the production of astaxanthin (Li et al., 2008). On the other hand, when a similar strategy was used involving mitochondrial respiration inhibitors, and a subsequent proteomic analysis using two-dimensional electrophoresis coupled to matrix assisted laser desorption-time-of-flight/time-of-flight tandem mass spectrometry (MALDI-TOF/TOF MS/MS), it was shown that in *Chlorella pyrenoidosa* cells, the production of some compounds such as lutein and chlorophyll also depends on the correct function of the mETC (Liu et al., 2018).

Recently, Uwizeye et al. (2021) performed a 3D microscopy morphometric analysis on seven different phytoplankton species. The results allowed to postulate that the phytoplankton subcellular topology is modulated by energy management constraints. In addition, lighting changes (*i.e.*, from low light to high light, a condition that was associated with stress production in microalgae) induced photosynthesis and respiration, increasing the occupation of the cell volume by mitochondria and also increasing the contact points between mitochondria and chloroplasts. In *Nannochloropsis* sp., for example, these structural changes were also associated with an increased respiratory and photosynthetic responses (Uwizeye et al., 2021). In addition, a recent work reported that the interaction and the energy exchange between chloroplasts and mitochondria increases the growth of microalgae and also the production of hydrogen, and that the molecular mechanism behind this particular phenotype would be related to the biogenesis of mitochondria (Elman et al., 2022).

**Figure 1:**
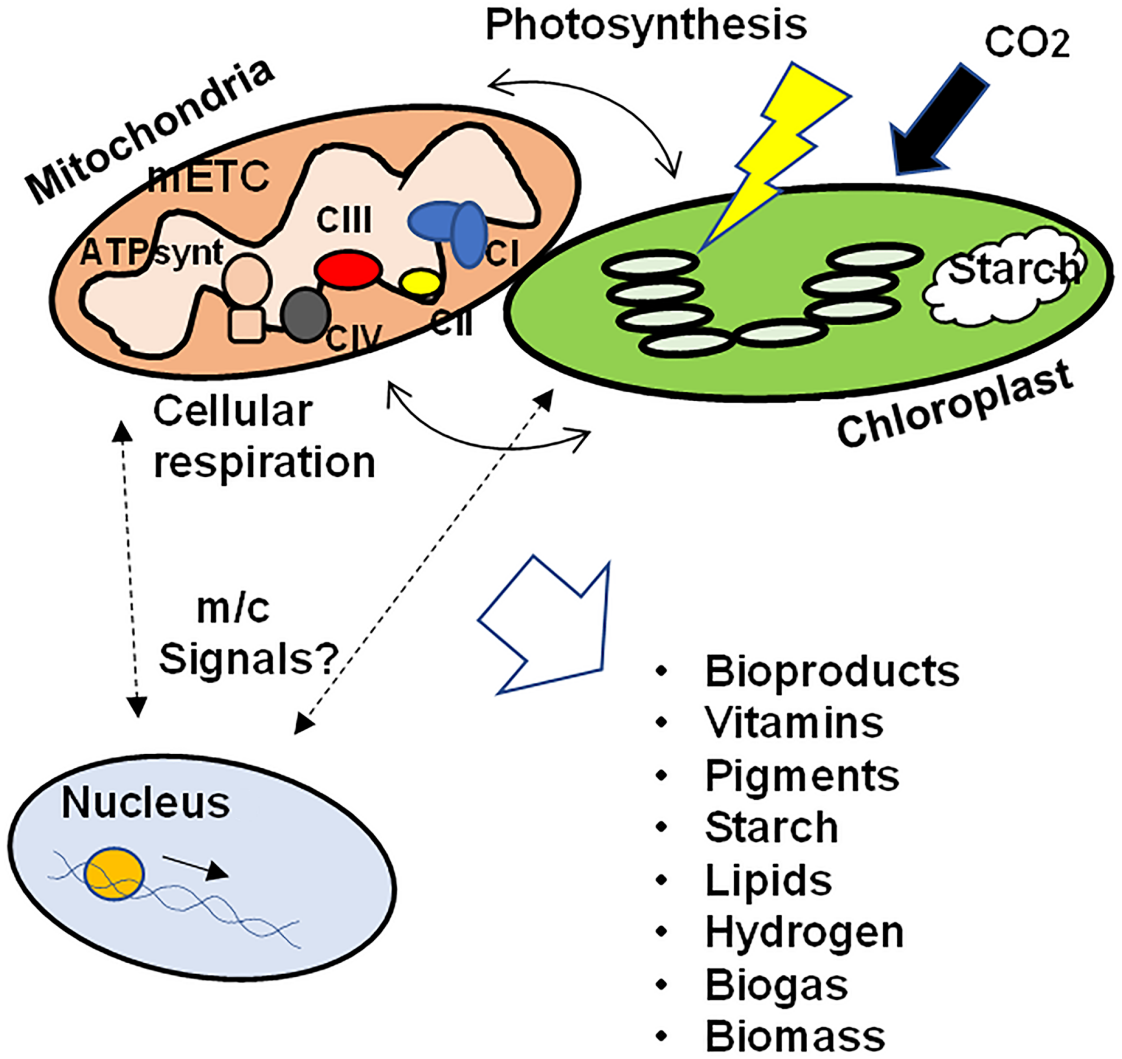
Schematic representation of the crosstalk between mitochondria and chloroplasts in microalgae. To ensure growth, biomass production, as well as the synthesis of different bioproducts, the correct function of the mETC, photosynthesis, the physical interaction between both organelles as well as the integration of retrograde signals between mitochondria/chloroplasts and the nuclear transcriptional response would be necessary. mETC, mitochondrial electron transport chain; CI-CIV, mitochondrial respiratory complexes I to IV; ATPsynt, mitochondrial ATP synthase; m/c signals, mitochondrial and/or chloroplastic signals.

These data support the hypothesis that the correct function of the mETC, the subcellular architecture, the contact points between mitochondria and chloroplasts, as well as the signals related to mitochondrial retrograde regulation between mitochondria and chloroplasts towards the nucleus are involved not only in the biosynthesis and accumulation of photosynthetic pigments in algae, but also in the biosynthesis of vitamins, bioproducts, starch, different lipids and in the production of hydrogen and biogas. In addition, the coordination of the functioning of the nucleus and the organelles would influence the adaptation to grow under different conditions, modulating the physiological and metabolic responses to maximize survival as well as biomass production (Fig. 1). Further studies are needed to elucidate the signals that regulate the crosstalk between mitochondria and chloroplasts and how these signals ultimately affect biomass generation and microalgae productivity, as well as the key metabolic pathways in the production of metabolites of interest.

### Engineering microalgae nuclear and organelle genomes

As we mentioned above, in a few years there will be a major challenge to produce high amounts of sustainable, cheap and environmentally friendly energy. A promising solution to overcome many issues in different areas such as agriculture, industry, medical sciences and energy production would be the development of design organisms (Cochrane et al., 2020). Synthetic biology has expanded the ability to enhance the genotype of an organism by the integration of optimized genes, leading to the selective improvement of different metabolic pathways and cellular phenotypes (Alper et al., 2010). Although there are various reports where the engineering of different microalgae was carried out by modifying the nuclear genome, little is known about the genetic modification of organelle genomes (Kumar et al., 2020).

In this sense, engineering genome organelles by introducing optimized genes would have several advantages over a nuclear transformation. First, genes are organized in polycistronic units, there is an absence of gene silencing and also reduced positional effects in organellar DNA (Alper et al., 2010; Cochrane et al., 2020). On the other hand, organelles allow metabolic pathways to be compartmentalized, which increases their efficiency and metabolic flow, and minimizes the competition of certain metabolites with other metabolic pathways located in other cellular compartments (Cochrane et al., 2020). The main goal of this strategy would be to modify the genomes of mitochondria and chloroplasts to improve them at a biochemical level, which could lead to greater productivity. Although there are numerous works where synthetic biology was applied in the design of other microorganisms of biotechnological interest, few work has been done related to the genetic modification of microalgae genomes. One of the main reasons would be that there is still little information of complete microalgae sequenced genomes compared to other microorganisms such as bacteria (Grama, Liu & Li, 2022; Kumar et al., 2020). However, in the last few years, several techniques have been developed that allowed the modification and editing of these genomes, however, the genetic engineering of algae was proved to be effective improving performance under laboratory conditions, but with limited success in industry.

Recently, a rapid and efficient method for engineering microalgae mitochondrial genomes was described. Cochrane et al. (2020) reported the use of the model organism *P. tricornutum*, which is a widely characterized diatom due to its ability to store high amounts of lipids for biofuel production. The described strategy includes a methodology to clone the complete chromosomes of microalgae inside *S. cerevisiae* and *E. coli*.

On the other hand, there are several reports where the nuclear transformation of different species of algae was carried out, such as *Chlamydomonas*, *Phaeodactylum*, *Dunaliella*, *Nannochloropsis* and *Haematococcus* (Kumar et al., 2020). Some of these reports allowed to improve the photosynthetic efficiency and biomass production, such as the overexpression of the RuBisCo activase (Wei et al., 2017), the fru-1,6-bisphosphate aldolase (Yang et al., 2017) or the engineering of the PSII D1 subunit in *Chlamydomonas* (Vinyard et al., 2014). Other works focused on the modification of several enzymes and metabolic pathways of lipid metabolism in algae, such as the expression of acetyl-CoA carboxylase, which encodes the enzyme that catalyzes the rate-limiting step in lipid synthesis, together with the malic enzyme, which catalyzes the production of pyruvate from malate. This led to an increase in the levels of accumulated lipids in *D. salina* (Talebi et al., 2014). A similar result was obtained after the overexpression of chloroplast diacylglycerol acyltransferase 3 in *P. tricornutum*, which also resulted in increased TAG levels in this algae (Niu et al., 2013) and also in *C. reinhardtii* (Carro et al., 2022). In addition, the overexpression of RuBisCo activase also increased TAG content in *N. oceanica* (Wei et al., 2017). Moreover, the possibility to generate knockout organisms by the CRISPR-Cas9 technique allowed the successful modification of different metabolic pathways. This new gene editing technology also drastically increased the possibility for design-driven synthetic biology (Kelterborn et al., 2022). These studies include the modification of phosphoenolpyruvate carboxylase 1 (PEPC1) in *C. reinhardtii* (Kao & Ng, 2017). PEPC is a cytosolic enzyme that catalyzes the formation of oxaloacetate which is subsequently converted to malate by malate dehydrogenase (MDH). This compound finally enters into the tricarboxylic acid (TCA) cycle to provide substrates for energy production in mitochondria. Thus, the deficiency of a cytosolic enzyme (PEPC1) causes a decrease in the efficiency of the mitochondrial TCA pathway with a redirection of the carbon flow towards lipid accumulation in *C. reinhardtii* (Deng et al., 2014; Kao & Ng, 2017). Another example of the modification of two enzymes encoded in the nucleus but with chloroplastic localization was the editing of zeaxanthin epoxidase (ZEP), which produce violaxanthin from zeaxanthin, and ADP-glucose pyrophosphorylase (AGP), which is the key enzyme in starch synthesis (Busi et al., 2014; Song et al., 2022). These gene modifications (PEPC1 and ZEP/AGP) produced an increase in lipid content in the *C. reinhardtii* mutants of about 30% and 81%, respectively.

Recently, it was described another interesting technique based on *Agrobacterium tumefaciens* transformation to improve lipid and terpenoid synthesis in *Schizochytrium* sp, a microorganism similar to marine microalgae which has become one of the most effective organisms used for the production of Omega-3 polyunsaturated fatty acids (Huang et al., 2021). The results showed that the overexpression of acetyl-CoA acetyltransferase produced an increase in the accumulation of terpenoid compounds, increasing the production of squalene, β-carotene and astaxanthin as well as the production of saturated fatty acids and polyunsaturated fatty acids. In addition, the deletion of three acyl-CoA oxidase genes also produced an increase in lipid content, demonstrating the usefulness of this strategy mediated by *A. tumefaciens* for the study of functional genes, as well as for the development of microalgae with higher productivity (Huang et al., 2021) (Table 1).

**Table 1:** Summary of the modifications that alter mitochondria and/or chloroplast function in microalgae. The table sumarizes the changes in phenotype after the modification of growth conditions or gene modification in different microalgae species.

Microalgae	Intervention	Modified traits and/or phenotype	References
*Phaeodactylum tricornutum*	LACS genes knockout/knockdown using CRISPR/Cas9	Deficiency in the accumulation of TAG and alteration in the fatty acid profile	Hao et al. (2022)
*Chlamydomonas reinhardtii*	Induction of mETC and chloroplast ETC by metabolites	Induction of photosynthetic genes	Matsuo & Obokata (2006)
*Chlorella zonfingiensis*	Growth in presence of glucose analogs and glucosamine (hexokinase inhibitor)	Increase in astaxanthin amounts that cause an increase in the levels of chloroplastic enzymes (β-carotenoid ketolase (BKT) and beta-carotenoid hydroxylase (CHYb))	Li et al. (2008)
	Antimycin A or rotenone	Decrease in β-carotenoid ketolase and β-carotenoid hydroxylase transcripts	Li et al. (2008)
	Salicyl hydroxamic acid	Decrease in astaxanthin amounts Inhibition of β-carotenoid ketolase and β-carotenoid hydroxylase transcripts	Li et al. (2008)
*Chlorella pyrenoidosa*	Antimycin A and sodium azide	Decrease in the production of lutein and chlorophyll	Liu et al. (2018)
	Salicyl hydroxamic acid	Increase biosynthesis of lutein and chlorophyll	Liu et al. (2018)
*Nannochloropsis oceanica*	Overexpression of RubisCo activase	Improvement of photosynthetic efficiency and biomass production. Increase TAG content	Wei et al. (2017)
*Nannochloropsis* sp.	Lighting changes (from low to high light)	Induction of photosynthesis and respiration, increase in the occupation of the cell volume by mitochondria and increase in the contacts between mitochondria and chloroplasts.	Uwizeye et al. (2021)
*Chlorella vulgaris*	Overexpression of fru-1,6-bisphosphate aldolase	Improvement of the photosynthetic efficiency and biomass production	Yang et al. (2017)
*Chlamydomonas reinhardtii*	Engineering of PSII D1 subunit	Improvement of the photosynthetic efficiency and biomass production	Vinyard et al. (2014)
*Dunaliella salina*	Expression of acetyl-CoA carboxylase and malic enzyme	Increase in lipid content	Talebi et al. (2014)
*Phaeodactylum tricornutum*	Overexpression of chloroplast diacylglycerol acyltransferase 3	Increase in TAG levels	Niu et al. (2013)
*Chlamydomonas reinhardtii*	Overexpression of chloroplast diacylglycerol acyltransferase 3	Increase in TAG levels	Carro et al. (2022)
*Chlamydomonas reinhardtii*	Mutation in the proton gradient regulation -5 protein (PGR5)	Deficiency in the cyclic electron flow Increase in respiration. Increase in hydrogen production	Elman et al. (2022)
*Chlamydomonas reinhardtii*	CRISPR/CAS9 modification of PEPC1 (deficiency of PEPC1)	Decrease in TCA pathway with an increase in lipid accumulation	Kao & Ng (2017)
*Chlamydomonas reinhardtii*	CRISPR/CAS9 modification of zeaxanthin epoxidase (ZEP) and ADPGlc PPase (AGP)	Increase in lipid content	Song et al. (2022)
*Schizochytrium* sp.	Overexpression of acetyl-CoA acetyltransferase (mediated by *A. tumefaciens* transformation)	Improvement of lipid and terpenoid synthesis	Huang et al. (2021)

Another interesting strategy would be to modify the levels of enzymes involved in glycolysis. It was reported that plant glycolytic enzymes are associated with the mitochondrial membrane to quickly direct the final products (pyruvate and/or malate) into mitochondria to continue their oxidation in the TCA cycle. For example, GAPC deficiency in plants showed a decrease in pyruvate levels and a slight increase in malate levels; however, the levels of TCA cycle intermediates decreased, as well as the expression of PEPC, which would lead to an increase in lipid accumulation (Rius et al., 2008). In the same direction, it was observed that the expression of a regulator of GAPC, SINAL7, which belongs from the SINA family proteins of E3 ubiquitin ligases, produced an increase in plant biomass, possibly associated with the decrease in GAPC activity or in GAPC association to the mitochondrial external membrane (Peralta et al., 2016, 2018, 2013). It remains to be evaluated whether the modification of glycolytic enzyme levels or the modification of GAPC regulation by this strategy would be useful to increase lipid accumulation and/or biomass in microalgae.

## Conclusions and future prospects

As we mentioned above, microalgae are microorganisms of high interest due to their ability to produce clean energy. In addition, there are numerous reports which described the use of these microorganisms for the production of different bio-products of biotechnological interest due to their metabolic diversity (Grama, Liu & Li, 2022; Kumar et al., 2020; Ng et al., 2017; Yang et al., 2017). In this way, the extensive use of microalgae could mitigate not only the problems related to energy deficiency, but also some of the problems caused by climate change and the feeding of the human population in the coming years.

The functions of mitochondria and chloroplasts are under the genetic control of the nuclear genome but also by their own genomes. These organelles are known to communicate with the nucleus and other compartments to maintain cellular homeostasis and integrate the cellular physiology. Thus, due to the fundamental role of both organelles in various key metabolic processes related with cell growth and development, it becomes especially important to carry out different genetic, molecular, biochemical and physiological studies on the role of these organelles in microalgae. For this, it is important to know more in detail the particular metabolism shown by the different algae species, the genes involved in organellar functioning and the regulation and signals involved in organelle retrograde signaling network. This knowledge will not only allow us to know more in detail the biology of microalgae, but also to have better molecular tools to modify these organisms to produce new strains that provide an environmental and biotechnological advantage such as a more efficient removal of pollutants in wastewater or their use for the production of clean energy and new bioproducts.

## Additional Information and Declarations

### Data Availability

The following information was supplied regarding data availability: This submission is a literature review.

## References

[ref-1] Alishah Aratboni H, Rafiei N, Garcia-Granados R, Alemzadeh A, Morones-Ramirez JR (2019). Biomass and lipid induction strategies in microalgae for biofuel production and other applications. Microbial Cell Factories.

[ref-2] Alper H, Cirino P, Nevoigt E, Sriram G (2010). Applications of synthetic biology in microbial biotechnology. Journal of Biomedicine and Biotechnology.

[ref-3] Banerjee C, Dubey KK, Shukla P (2016). Metabolic engineering of microalgal based biofuel production: prospects and challenges. Frontiers in Microbiology.

[ref-4] Busi MV, Barchiesi J, Martin M, Gomez-Casati DF (2014). Starch metabolism in green algae. Starch.

[ref-5] Busi MV, Gomez-Casati DF (2012). Exploring frataxin function. IUBMB Life.

[ref-6] Busi MV, Gomez-Lobato ME, Araya A, Gomez-Casati DF (2011). Mitochondrial dysfunction affects chloroplast functions. Plant Signaling & Behavior.

[ref-7] Carro MLM, Gonorazky G, Soto D, Mamone L, Bagnato C, Pagnussat LA, Beligni MV (2022). Expression of Chlamydomonas reinhardtii chloroplast diacylglycerol acyltransferase 3 is induced by light in concert with triacylglycerol accumulation. The Plant Journal.

[ref-8] Chen CY, Kao AL, Tsai ZC, Chow TJ, Chang HY, Zhao XQ, Chen PT, Su HY, Chang JS (2016). Expression of type 2 diacylglycerol acyltransferse gene DGTT1 from Chlamydomonas reinhardtii enhances lipid production in Scenedesmus obliquus. Biotechnology Journal.

[ref-9] Cochrane RR, Brumwell SL, Soltysiak M, Hamadache S, Davis JG, Wang J, Tholl SQ, Janakirama P, Edgell DR, Karas BJ (2020). Rapid method for generating designer algal mitochondrial genomes. Algal Research.

[ref-10] Deng X, Cai J, Li Y, Fei X (2014). Expression and knockdown of the PEPC1 gene affect carbon flux in the biosynthesis of triacylglycerols by the green alga Chlamydomonas reinhardtii. Biotechnology Letters.

[ref-11] Du ZY, Benning C (2016). Triacylglycerol accumulation in photosynthetic cells in plants and algae. Subcellular Biochemistry.

[ref-12] Elman T, Ho TT, Milrad Y, Hippler M, Yacoby I (2022). Enhanced chloroplast-mitochondria crosstalk promotes ambient algal-H2 production. Cell Reports Physical Science.

[ref-13] Gomez-Casati DF, Busi MV, Barchiesi J, Pagani MA, Marchetti-Acosta NS, Terenzi A (2021). Fe-S protein synthesis in green algae mitochondria. Plants (Basel).

[ref-14] Goold H, Beisson F, Peltier G, Li-Beisson Y (2015). Microalgal lipid droplets: composition, diversity, biogenesis and functions. Plant Cell Reports.

[ref-15] Goold HD, Cuine S, Legeret B, Liang Y, Brugiere S, Auroy P, Javot H, Tardif M, Jones B, Beisson F, Peltier G, Li-Beisson Y (2016). Saturating light induces sustained accumulation of oil in plastidal lipid droplets in Chlamydomonas reinhardtii. Plant Physiology.

[ref-16] Grama SB, Liu Z, Li J (2022). Emerging trends in genetic engineering of microalgae for commercial applications. Marine Drugs.

[ref-17] Gray MW (1999). Evolution of organellar genomes. Current Opinion in Genetics & Development.

[ref-18] Gross J, Bhattacharya D (2009). Mitochondrial and plastid evolution in eukaryotes: an outsiders’ perspective. Nature Reviews Genetics.

[ref-19] Guerin M, Huntley ME, Olaizola M (2003). Haematococcus astaxanthin: applications for human health and nutrition. Trends in Biotechnology.

[ref-20] Hao X, Chen W, Amato A, Jouhet J, Marechal E, Moog D, Hu H, Jin H, You L, Huang F, Moosburner M, Allen AE, Gong Y (2022). Multiplexed CRISPR/Cas9 editing of the long-chain acyl-CoA synthetase family in the diatom Phaeodactylum tricornutum reveals that mitochondrial ptACSL3 is involved in the synthesis of storage lipids. New Phytologist.

[ref-21] Harmon J, Findinier J, Ishii NT, Herbig M, Isozaki A, Grossman A, Goda K (2022). Intelligent image-activated sorting of Chlamydomonas reinhardtii by mitochondrial localization. Cytometry Part A.

[ref-22] Huang PW, Xu YS, Sun XM, Shi TQ, Gu Y, Ye C, Huang H (2021). Development of an efficient gene editing tool in Schizochytrium sp. and improving its lipid and terpenoid biosynthesis. Frontiers in Nutrition.

[ref-23] Jennings MJ, Hathazi D, Nguyen CDL, Munro B, Munchberg U, Ahrends R, Schenck A, Eidhof I, Freier E, Synofzik M, Horvath R, Roos A (2021). Intracellular lipid accumulation and mitochondrial dysfunction accompanies endoplasmic reticulum stress caused by loss of the co-chaperone DNAJC3. Frontiers in Cell and Developmental Biology.

[ref-24] Kao PH, Ng IS (2017). CRISPRi mediated phosphoenolpyruvate carboxylase regulation to enhance the production of lipid in Chlamydomonas reinhardtii. Bioresource Technology.

[ref-25] Kelterborn S, Boehning F, Sizova I, Baidukova O, Evers H, Hegemann P, Zurbriggen MD (2022). Gene editing in green alga Chlamydomonas reinhardtii via CRISPR-Cas9 ribonucleoproteins. Plant Synthetic Biology.

[ref-26] Kotiadis VN, Duchen MR, Osellame LD (2014). Mitochondrial quality control and communications with the nucleus are important in maintaining mitochondrial function and cell health. Biochimica et Biophysica Acta.

[ref-27] Kumar G, Shekh A, Jakhu S, Sharma Y, Kapoor R, Sharma TR (2020). Bioengineering of microalgae: recent advances, perspectives, and regulatory challenges for industrial application. Frontiers in Bioengineering and Biotechnology.

[ref-28] Kwak HS, Kima JYH, Woo HM, Jin E, Min BK, Sima SJ (2016). Synergistic effect of multiple stress conditions for improving microalgal lipid production. Algal Research.

[ref-29] Lee SJ, Zhang J, Choi AM, Kim HP (2013). Mitochondrial dysfunction induces formation of lipid droplets as a generalized response to stress. Oxidative Medicine and Cellular Longevity.

[ref-30] Li Y, Han D, Hu G, Dauvillee D, Sommerfeld M, Ball S, Hu Q (2010). Chlamydomonas starchless mutant defective in ADP-glucose pyrophosphorylase hyper-accumulates triacylglycerol. Metabolic Engineering.

[ref-31] Li Y, Huang J, Sandmann G, Chen F (2008). Glucose sensing and the mitochondrial alternative pathway are involved in the regulation of astaxanthin biosynthesis in the dark-grown Chlorella zofingiensis (Chlorophyceae). Planta.

[ref-32] Lill R, Muhlenhoff U (2008). Maturation of iron-sulfur proteins in eukaryotes: mechanisms, connected processes, and diseases. Annual Review of Biochemistry.

[ref-33] Liu Z, Butow RA (2006). Mitochondrial retrograde signaling. Annual Review of Genetics.

[ref-34] Liu ZH, Li T, He QY, Sun Z, Jiang Y (2018). Role of mitochondria in regulating lutein and chlorophyll biosynthesis in Chlorella pyrenoidosa under heterotrophic conditions. Marine Drugs.

[ref-35] Matsuo M, Obokata J (2006). Remote control of photosynthetic genes by the mitochondrial respiratory chain. The Plant Journal.

[ref-36] Moller IM, Rasmusson AG, Van Aken O (2021). Plant mitochondria—past, present and future. The Plant Journal.

[ref-37] Nath K, Najafpour MM, Voloshin RA, Balaghi SE, Tyystjarvi E, Timilsina R, Eaton-Rye JJ, Tomo T, Nam HG, Nishihara H, Ramakrishna S, Shen JR, Allakhverdiev SI (2015). Photobiological hydrogen production and artificial photosynthesis for clean energy: from bio to nanotechnologies. Photosynthesis Research.

[ref-38] Ng IS, Tan SI, Kao PH, Chang YK, Chang JS (2017). Recent developments on genetic engineering of microalgae for biofuels and bio-based chemicals. Biotechnology Journal.

[ref-39] Niu YF, Zhang MH, Li DW, Yang WD, Liu JS, Bai WB, Li HY (2013). Improvement of neutral lipid and polyunsaturated fatty acid biosynthesis by overexpressing a type 2 diacylglycerol acyltransferase in marine diatom Phaeodactylum tricornutum. Marine Drugs.

[ref-40] Peralta DA, Araya A, Busi MV, Gomez-Casati DF (2016). The E3 ubiquitin-ligase SEVEN IN ABSENTIA like 7 mono-ubiquitinates glyceraldehyde-3-phosphate dehydrogenase 1 isoform in vitro and is required for its nuclear localization in Arabidopsis thaliana. The International Journal of Biochemistry & Cell Biology.

[ref-41] Peralta DA, Araya A, Gomez-Casati DF, Busi MV (2018). Over-expression of SINAL7 increases biomass and drought tolerance, and also delays senescence in Arabidopsis. Journal of Biotechnology.

[ref-42] Peralta DA, Araya A, Nardi CF, Busi MV, Gomez-Casati DF (2013). Characterization of the Arabidopsis thaliana E3 ubiquitin-ligase AtSINAL7 and identification of the ubiquitination sites. PLOS ONE.

[ref-43] Poyton RO, McEwen JE (1996). Crosstalk between nuclear and mitochondrial genomes. Annual Review of Biochemistry.

[ref-44] Raghavendra AS, Padmasree K (2003). Beneficial interactions of mitochondrial metabolism with photosynthetic carbon assimilation. Trends in Plant Science.

[ref-45] Rius SP, Casati P, Iglesias AA, Gomez-Casati DF (2008). Characterization of Arabidopsis lines deficient in GAPC-1, a cytosolic NAD-dependent glyceraldehyde-3-phosphate dehydrogenase. Plant Physiology.

[ref-46] Ryan MT, Hoogenraad NJ (2007). Mitochondrial-nuclear communications. Annual Review of Biochemistry.

[ref-47] Skjanes K, Rebours C, Lindblad P (2013). Potential for green microalgae to produce hydrogen, pharmaceuticals and other high value products in a combined process. Critical Reviews in Biotechnology.

[ref-48] Song I, Kim S, Kim J, Oh H, Jang J, Jeong SJ, Baek K, Shin WS, Sim SJ, Jin E (2022). Macular pigment-enriched oil production from genome-edited microalgae. Microbial Cell Factories.

[ref-49] Sun H, Zhao W, Mao X, Li Y, Wu T, Chen F (2018). High-value biomass from microalgae production platforms: strategies and progress based on carbon metabolism and energy conversion. Biotechnology for Biofuels.

[ref-50] Talebi AF, Tohidfar M, Bagheri A, Lyon SR, Salehi-Ashtiani K, Tabatabaei M (2014). Manipulation of carbon flux into fatty acid biosynthesis pathway in Dunaliella salina using AccD and ME genes to enhance lipid content and to improve produced biodiesel quality. Biofuel Research Journal.

[ref-51] Turchi R, Tortolici F, Guidobaldi G, Iacovelli F, Falconi M, Rufini S, Faraonio R, Casagrande V, Federici M, De Angelis L, Carotti S, Francesconi M, Zingariello M, Morini S, Bernardini R, Mattei M, La Rosa P, Piemonte F, Lettieri-Barbato D, Aquilano K (2020). Frataxin deficiency induces lipid accumulation and affects thermogenesis in brown adipose tissue. Cell Death & Disease.

[ref-52] Uwizeye C, Decelle J, Jouneau PH, Flori S, Gallet B, Keck JB, Bo DD, Moriscot C, Seydoux C, Chevalier F, Schieber NL, Templin R, Allorent G, Courtois F, Curien G, Schwab Y, Schoehn G, Zeeman SC, Falconet D, Finazzi G (2021). Morphological bases of phytoplankton energy management and physiological responses unveiled by 3D subcellular imaging. Nature Communications.

[ref-53] Vinyard DJ, Gimpel J, Ananyev GM, Mayfield SP, Dismukes GC (2014). Engineered Photosystem II reaction centers optimize photochemistry versus photoprotection at different solar intensities. Journal of the American Chemical Society.

[ref-54] Vitova M, Bisova K, Kawano S, Zachleder V (2015). Accumulation of energy reserves in algae: from cell cycles to biotechnological applications. Biotechnology Advances.

[ref-55] Wei L, Wang Q, Xin Y, Lu Y, Xu J (2017). Enhancing photosynthetic biomass productivity of industrial oleaginous microalgae by overexpression of RuBisCO activase. Algal Research.

[ref-56] Yang B, Liu J, Ma X, Guo B, Liu B, Wu T, Jiang Y, Chen F (2017). Genetic engineering of the Calvin cycle toward enhanced photosynthetic CO_2_ fixation in microalgae. Biotechnology for Biofuels.

